# Reliability of new software in measuring cervical multifidus diameters and shoulder muscle strength in a synchronized way; an ultrasonographic study

**DOI:** 10.1590/bjpt-rbf.2014.0097

**Published:** 2015-09-01

**Authors:** Leila Rahnama, Asghar Rezasoltani, Minoo Khalkhali-Zavieh, Behnam Rahnama, Farhang Noori-Kochi

**Affiliations:** 1 Department of Physical Therapy, University of Social Welfare and Rehabilitation Sciences, Tehran, Iran; 2 Physiotherapy Research Center, Faculty of Rehabilitation Sciences, Shahid Beheshti University of Medical Sciences, Tehran, Iran; 3 Department of Physical Therapy, Faculty of Rehabilitation Sciences, Shahid Beheshti University of Medical Sciences, Tehran, Iran; 4 Department of Computer Science, Engineering, and IT, Shiraz University, Shiraz, Iran; 5 Department of Radiology, Medical Imaging Research Center, Shiraz, Iran

**Keywords:** software, cervical multifidus muscle, neck pain, ultrasonography, reliability

## Abstract

**OBJECTIVES:**

This study was conducted with the purpose of evaluating the inter-session reliability of new software to measure the diameters of the cervical multifidus muscle (CMM), both at rest and during isometric contractions of the shoulder abductors in subjects with neck pain and in healthy individuals.

**METHOD:**

In the present study, the reliability of measuring the diameters of the CMM with the Sonosynch software was evaluated by using 24 participants, including 12 subjects with chronic neck pain and 12 healthy individuals. The anterior-posterior diameter (APD) and the lateral diameter (LD) of the CMM were measured in a resting state and then repeated during isometric contraction of the shoulder abductors. Measurements were taken on separate occasions 3 to 7 days apart in order to determine inter-session reliability. Intraclass correlation coefficient (ICC), standard error of measurement (SEM), and smallest detectable difference (SDD) were used to evaluate the relative and absolute reliability, respectively.

**RESULTS:**

The Sonosynch software has shown to be highly reliable in measuring the diameters of the CMM both in healthy subjects and in those with neck pain. The ICCs 95% CI for APD ranged from 0.84 to 0.94 in subjects with neck pain and from 0.86 to 0.94 in healthy subjects. For LD, the ICC 95% CI ranged from 0.64 to 0.95 in subjects with neck pain and from 0.82 to 0.92 in healthy subjects.

**CONCLUSIONS:**

Ultrasonographic measurement of the diameters of the CMM using Sonosynch has proved to be reliable especially for APD in healthy subjects as well as subjects with neck pain.

## Introduction

Real-time ultrasound (US) imaging is frequently used to evaluate muscle activity^1^^-^ 3. It has the advantage of being an accessible, inexpensive, yet reliable and valid method of measuring muscle diameters both at rest and in contraction^1^^,^ 2. Therefore, it has become a generally acceptable technique used to assess muscle activity indirectly^4^^-^ 9. The reliability of US measurements of muscle diameters has already been established for deep neck muscles^4^^,^ 7 ^,^ 8 ^,^ 10, lumbar muscles^11^^-^ 13, and abdominal muscles^5^^,^ 14. During the past decade, ultrasonography has been increasingly employed to indirectly evaluate deep cervical muscle activity as an alternative tool to the costly MRI^15^^-^ 18 when assessing the activation of these muscles.

Kristjansson^7^ reported the ultrasonography protocol for detecting the size of the cervical multifidus muscle (CMM) as a reliable method; however, its reliability for individuals with neck pain was reported only at an acceptable level^7^. Lin et al.^8^ evaluated the reliability of deep dorsal neck muscle measurements at the level of C4 both at rest and contracted. They reported that ultrasonography was a highly reliable method of measuring the thickness of upper dorsal cervical muscles both at rest and when contracted^8^. Lee et al.^10^ assessed the reliability of ultrasonography of the cervical multifidus muscle both at rest and contracted and found it to be a reliable method to measure the thickness of the cervical multifidus muscle in healthy subjects.

However, an important problem exists regarding the practical use of ultrasonography. There is a need to freeze an image and stop the procedure to allow the measurement of the muscle diameters or amount of muscle force at a particular time. This limitation makes researchers unable to appraise the muscle diameter at different states of the contraction period without interruptions to the procedure. The Sonosynch software that was developed to overcome this limitation has the capability of simultaneously detecting and recording US images and force data from the muscle for offline measurements. Therefore, the aim of this study was to assess the reliability of the measurement of the CMM diameter as a sample muscle in a rested state and during the isometric contraction of shoulder abductors using the Sonosynch software.

## Method

### Participants

A total of 24 individuals, including 12 healthy males (mean age 27.45±4.37, mean BMI 23.28±1.67) and 12 males with chronic neck pain (mean age 28.90±5.53, mean BMI 23.44±1.59) voluntarily participated in this study. Any history of previous spinal surgery, congenital deformity, neck or back trauma, inflammatory diseases like rheumatoid arthritis were considered exclusion criteria. Participants with neck pain had to have experienced neck pain for at least 3 months in the last year. A full explanation of the impending procedure was given to all participants before giving their informed consent, followed by a practice of three random trials of the procedure in order to familiarize themselves with it. The Ethical Board of the Physical Therapy Research Center, Shahid Beheshti University of Medical Sciences, Tehran, Iran, approved the study procedure (approval number 1391-1-144-1058).

## Procedure

### Recording isometric force

**Figure 1.**
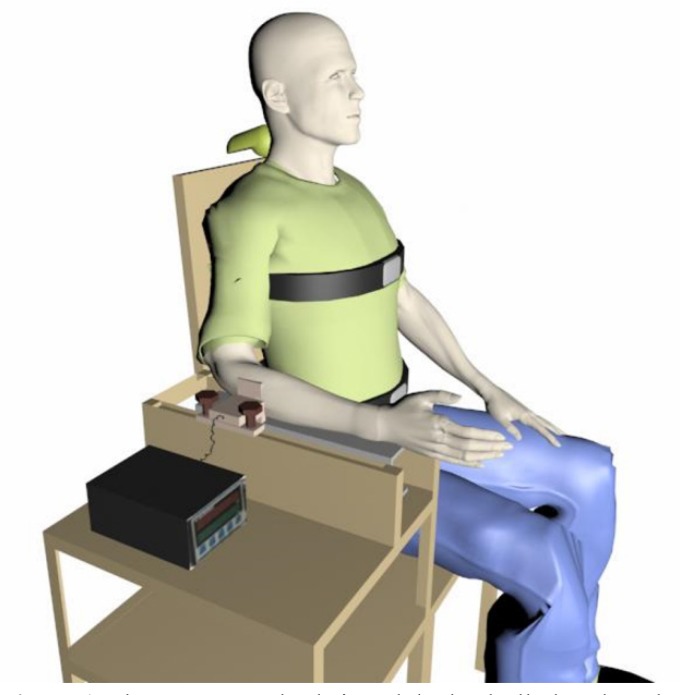
The custom made chair and the loadcell placed on the U shaped device to record shoulder muscle isometric contraction.

A ZEMIC load cell model H3-C3-100 Kg-3B was placed on a U-shaped device located on the right armrest of a custom-made chair. This chair was designed to record the isometric forces of the shoulder muscles. The U-shaped device was placed on the right armrest of the chair to let the load cell move. This design allowed the examiner to adjust the load cell position to various anthropometric measures or record different force directions. The expected direction for this study was abduction. Participants were instructed to sit on the chair, keeping their heads neutral, put their right forearms on the armrest, and gradually apply force against the load cell toward shoulder abduction (Figure 1). It has been reported that isometric contractions of shoulder muscles cause CMM contraction, providing stability to the cervical spine. Therefore, participants were told to contract their shoulder abductors so that changes in the thickness of the CMM could be evaluated using the Sonosynch software^19^. Then, they were asked to reach their maximal voluntary contraction (MVC) in 10 seconds. Three trials of MVC were done 60 - secs apart. The trial with the maximum amount of MVC was chosen for data analysis and measurement of CMM thickness^16^.

The trial was performed three times to ensure the subject reached the maximum possible MVC rather than calculating their average.

### Ultrasound imaging

US imaging of the CMM was performed using an ultrasound device (Accuvix V20 prestige, Samsung Medison, Korea) with an 8 MHz, 4.5 cm linear array transducer. To measure CMM thickness, the spinous process of C4 was palpated. To confirm the spinal level, ultrasonography guidance was used^10^. C4 was chosen as it is claimed that CMM is easy to measure at this level^7^. Further explanation is found in the Discussion section. Next, the examiner placed the transducer horizontally on the right side of the C4 spinous process. Then the transducer was tilted slightly upward or downward to see the echogenic lamina and the interfacing fascia clearly. At this level, the CMM was seen lateral to the spinous process, rotator muscle and laminar junction, medial to the articular process and just under the fascia of the semispinalis cervicis muscle^10^. Images were taken firstly at rest and then during the isometric contraction of the shoulder abductors until participants reached their MVCs within the given 10 seconds. Anterior posterior diameter (APD) or the thickness of the CMM was measured as the longest distance between the lamina and the interfacing fascia of semispinalis cervicis.

### Software

**Figure 2.**
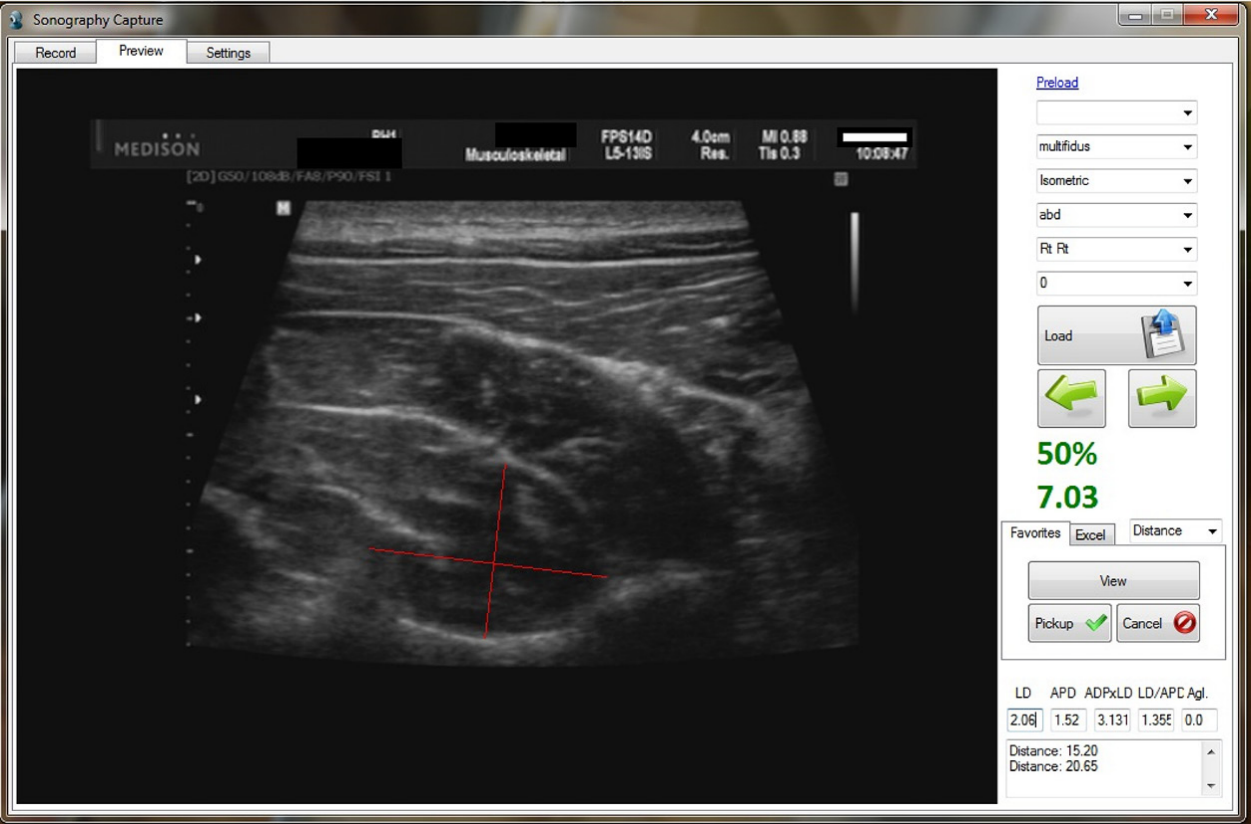
Sonosynch Software Interface

The offline measurement of the CMM thickness at rest and at different states of isometric contraction of the shoulder abductor muscles was carried out by the Sonosynch software. This software picked up and stored the US images in addition to the load cell data with a sampling rate of 20ps. This capability enables us to appraise muscle diameters both at rest and at different desired states of MVC. Sonosynch captures muscle forces from the state of rest to 100% MVC. Therefore, the examiner was able to measure the CMM thickness in every desirable amount of MVC. On average, 200 images corresponding to their force level can be stored in 10 seconds. Maximum force is considered as 100% MVC. Having all values with a high sampling rate allows us to choose any value between 0 to 100% MVC (Figure 2). In the present study, we assessed CMM thickness at rest (0%) and at 25%, 50%, 75%, and 100% MVC of isometric contraction of shoulder abductors obtained from the trials with higher MVCs^19^.

### Reliability study

To evaluate the inter-session reliability for measuring the thickness and lateral diameter of the CMM as well as shoulder abductor strength captured and stored by the software, the procedure was out by the same rater on two separate days, three to seven days apart. The entire procedure was completed on both occasions and in both groups.

### Statistical analysis

To estimate the relative reliability, a two-way mixed model of Intraclass correlation coefficient (ICC) with ICC_3,1_ was carried out. For the ICC, a 95% of confidence interval (CI) was reported in order to indicate the precision of estimates. To define the absolute reliability, standard error of measurements (SEM) and the smallest detectable difference (SDD) were computed. SEM was measured as the square root of the mean square error term derived from analysis of variance^20^ and SDD was defined as 95% CI of SEM, calculated as 1.96 SEM^4^^,^ 21. The level of significance defined as p<0.05.

## Results

According to Munro's classification for reliability coefficients^22^, we found a high to very high level of reliability with ICC ranging from 0.84 to 0.94, SEM ranging from 0.01 to 0.09, and SDD ranging from 0.03 to 0.25 for the APD and ICC ranging from 0.64 to 0.95, SEM ranging from 0.03 to 0.11, and SDD ranging from 0.08 to 0.30 for the LD. The only exception for the abovementioned results was regarding the reliability of LD in 100% MVC of shoulder abductors which showed a moderate correlation, with ICC 0.64, SEM 0.14, and SDD 0.39 (Tables 1 and 2).

**Table 1.** Inter-session reliability of the measurement of anterior-posterior dimension (APD) of the cervical multifidus muscle using the Sonosynch software.

APD		Healthy subjects			CNP subjects	
MVC %	ICC	SEM	SDD	ICC	SEM	SDD
0	0.89	0.05	0.14	0.84	0.01	0.03
25	0.88	0.06	0.17	0.88	0.04	0.11
50	0.86	0.07	0.19	0.94	0.03	0.08
75	0.94	0.04	0.11	0.94	0.03	0.8
100	0.87	0.09	0.25	0.91	0.04	0.11

MVC: Maximal Voluntary Contraction; ICC: Intraclass Correlation Coefficient; SEM: Standard Error of Measurement; SDD: Smallest Detectable Difference; CNP: Chronic neck pain.

**Table 2.** Inter-session Reliability of the measurement of Lateral dimension (LD) of the cervical multifidus muscle using the Sonosynch software.

LD		Healthy subjects			CNP subjects	
	ICC	SEM	SDD	ICC	SEM	SDD
0	0.92	0.06	0.17	0.83	0.06	0.17
25	0.82	0.11	0.30	0.95	0.04	0.11
50	0.88	0.07	0.19	0.87	0.06	0.17
75	0.89	0.03	0.08	0.82	0.07	0.19
100	0.92	0.07	0.19	0.64	0.14	0.39

MVC: Maximal Voluntary Contraction; ICC: Intraclass Correlation Coefficient; SEM: Standard Error of Measurement; SDD: Smallest Detectable Difference; CNP: Chronic neck pain.

A very high reliability, with ICC ranging from 0.81 to 0.91, SEM ranging from 0.31 to 1.10, and SDD ranging from 0.86 to 3.05 (Table 3), was found for the inter-session reliability of shoulder abductor strength from 25% to 100% MVC.

**Table 3.** Inter-session reliability of the measurement of shoulder muscle strength using the Sonosynch software.

Force		Healthy subjects			CNP subjects	
MVC %	ICC	SEM	SDD	ICC	SEM	SDD
25	0.81	0.42	1.16	0.88	0.31	0.86
50	0.86	0.75	2.08	0.91	0.55	1.52
75	0.84	0.47	1.30	0.91	0.84	2.33
100	0.84	0.62	1.72	0.91	1.10	3.05

MVC: Maximal Voluntary Contraction; ICC: Intraclass Correlation Coefficient; SEM: Standard Error of Measurement; SDD: Smallest Detectable Difference; CNP: Chronic neck pain.

## Discussion

The results of the present study showed that all measurements of the muscle's diameters and strength conducted by the Sonosynch software had high to very high inter-session reliability except for the lateral diameter of the cervical multifidus in 100% of MVC of shoulder abductors which was shown to have moderate reliability.

We found a high inter-session reliability of APD and LD measurement of the CMM both at rest and contracted. However, Rankin et al.^23^reported a very high inter-session reliability (with ICC ranging from 0.98 to 0.99) for ultrasonographic measurement of deep dorsal neck muscles. In contrast, Kristjansson^7^ reported moderate to acceptable reliability when measuring the size of CMM in healthy individuals. This discrepancy between the results may be due to the fact that Rankin et al.^23^reported the CSA of CMM and semispinalis cervicis as one muscle. In the present study, we measured the APD and LD of the CMM separate from the semispinalis cervicis muscle. To the best of our knowledge, it is the first study to attempt to establish a new software that simultaneously records and measures muscle diameters and strength conducted by a US device and load cell respectively in both healthy subjects and patients with neck pain. This software enables researchers to record US images while their subjects are doing the contractile task and to process them offline. The high reliability of measuring the diameters of the CMM and shoulder abductor strength shown in the present study encourages widespread usage of this software in studies aiming to evaluate activity of the muscles during functional daily tasks.

The only exception for the above-mentioned results of the present study is a moderate inter-session reliability of CMM lateral diameter at 100% of shoulder abductor MVC in patients with neck pain. Kristjansson also reported a good reliability of ultrasonographic measurement of CMM diameters in healthy individuals but not in patients with neck pain^7^. The possible explanation for such a result may possibly be due to the position that participants took to produce the maximum abductor force in addition to the fact that recognizing interfacing muscle fascia in patients is more difficult than in healthy individuals^10,24^. Lee et al.^10^ also argued that the anatomical structure of the CMM causes the lateral boundaries to be less distinguishable in ultrasonography. In fact, the CMM comes from the spinous process of the lower cervical vertebra and attaches to the articular process of the upper process. Considering this fact, the axial resolution of ultrasonography is better than its horizontal resolution, therefore, it is more precise to measure its APD relative to the LD^16^.

We also found a very low SEM and SDD for both APD and LD, which strengthens the ability to detect the diameters of CMM especially for APD with ultrasonography. This means that we require an average of 10% change in the APD to be detected by the Sonosynch software, which is in line with previous studies^4^^,^ 25.

Regarding the inter-session reliability of MVCs, we found a very high ICC in both healthy individuals and patients with neck pain. These results are higher than those reported by Cadogan et al.^26^ and Celik at al.^27^. This disagreement may be due to using different devices for recording the muscle strength. However, our results support the findings of Adsuar et al.^28^ who reported a moderate to very high relative reliability for the measurement of isotonic strength of shoulder muscles^28^.

We decided to evaluate the CMM thickness at the level of C4. The CMM is easily detected at this level^7^. However, the CMM thickness has been measured in other cervical levels as well^10^^,^ 29.

### Limitations and future studies

There may be a few limitations to generalizing the accomplishments of this research to all patients suffering from neck pain due to our focus on only subjects with chronic neck pain. Therefore, future study is recommended to evaluate the inter-session reliability of the software on subjects with other types of neck pain, such as those with whiplash injury. In this research, we measured the CMM diameters as well as shoulder abductor muscle strength. However, future reliability assessment of the software for measuring other muscle diameters and strengths is recommended to expand the use of this software to other cases.

In conclusion, the inter-session reliability of the Sonosynch software is high when tested by one examiner. This software provides the capability of capturing and saving the US images and load cell data in a synchronized way to allow offline measurements of muscle diameters during the muscle contraction period.
